# Effect on Different Glial Cell Types of S100B Modulation in Multiple Sclerosis Experimental Models

**DOI:** 10.3390/ijms26135948

**Published:** 2025-06-20

**Authors:** Maria De Carluccio, Gabriele Di Sante, Maria Elisabetta Clementi, Mariangela Ruggirello, Anna Maria Stabile, Alessandra Pistilli, Stefano Marini, Vincenzo Romano Spica, Mario Rende, Francesco Ria, Fabrizio Michetti

**Affiliations:** 1 Department of Neuroscience, Università Cattolica del Sacro Cuore, Largo Francesco Vito 1, 00168 Rome, Italy; maria.decarluccio@unicatt.it; 2 Department of Surgery and Medicine, Institute of Human, Clinical and Forensic Anatomy, P.le L Severi 1, 06132 Perugia, Italy; gabriele.disante@unipg.it (G.D.S.); mariangela.ruggirello@unicatt.it (M.R.); anna.stabile@unipg.it (A.M.S.); alessandra.pistilli@unipg.it (A.P.); mario.rende@unipg.it (M.R.); 3 Istituto di Scienze e Tecnologie Chimiche “Giulio Natta” SCITEC-CNR, Largo F. Vito 1, 00168 Rome, Italy; elisabetta.clementi@scitec.cnr.it; 4 Department of Translational Medicine and Surgery, Section of General Pathology, Università Cattolica del Sacro Cuore, Largo Francesco Vito 1, 00168 Rome, Italy; francesco.ria@unicatt.it; 5 Department of Clinical Sciences and Translational Medicine, Università of Rome “Tor Vergata”, Via Montpellier 1, 00133 Rome, Italy; stefano.marini@uniroma2.it; 6 Department of Movement, Human and Health Sciences, Laboratory of Epidemiology and Biotechnologies, University of Rome “Foro Italico”, Piazza Lauro De Bosis 6, 00135 Rome, Italy; vincenzo.romanospica@uniroma4.it; 7 Department of Laboratory and Hematological Sciences, Fondazione Policlinico Universitario A. Gemelli IRCCS, Largo Agostino Gemelli 1-8, 00168 Rome, Italy; 8 Department of Medicine, “Libera Università Mediterranea” University, 70010 Casamassima, Italy; 9 Genes, Via Venti Settembre 118, 00187 Rome, Italy

**Keywords:** S100B, arundic acid, pentamidine isethionate, experimental autoimmune encephalomyelitis, multiple sclerosis

## Abstract

It has been demonstrated that S100B actively participates in neuroinflammatory processes of different diseases of the central nervous system (CNS), such as experimental autoimmune encephalomyelitis (EAE), a recognized animal model for multiple sclerosis (MS). The inhibition of S100B activity using pentamidine and of S100B synthesis using arundic acid are able to determine an amelioration of the clinical and pathologic parameters of MS with milder and delayed symptoms. This study further goes in detail on the role of S100B, and in particular of astrocytic S100B, in these neuroinflammatory processes. To this aim, we used a model of S100B knockout (KO) mice. As expected, S100B protein levels were significantly reduced in the S100B KO mouse strain resulting in an amelioration of clinical and pathological parameters (clinical and morphological analyses). To dissect the potential mechanisms that could explain the role of S100B in the development of EAE, we sorted, cultured, and compared glial subpopulations (astrocytes, oligodendrocytes, and microglia) derived from S100B KO and wild type mice, through flow cytometric panels and ELISA. Glial cells were analyzed for proinflammatory molecules showing a significant reduction of TNFα protein in mice where S100B was silenced. To dissect the role of S100B in MS, we cultured astrocytes and microglial cells magnetically sorted and enriched from the brains of EAE-affected animals, both from KO and wild type animals. Both genetic silencing of S100B and pharmacological inhibition with S100B-targeting compounds demonstrated a direct impact on specific subpopulations of astrocytes (mainly), oligodendrocytes, and microglia. The present results further individuate astrocytic S100B as a key factor and as a potential therapeutic target for EAE neuroinflammatory processes.

## 1. Introduction

Reactive astrocytosis and oligodendrocyte dysfunction are central features in the pathogenesis of multiple sclerosis (MS), contributing significantly to demyelination, neurodegeneration, and failed repair mechanisms. Reactive astrocytes, which become hypertrophic and proliferative in response to CNS injury, secrete a range of pro-inflammatory cytokines, chemokines, and neurotoxic molecules such as glutamate and also S100B [1,2]. While astrocytes can initially limit damage by maintaining blood–brain barrier integrity and modulating immune cell infiltration, chronic astrocytosis exacerbates inflammation, fosters glial scar formation, and inhibits oligodendrocyte progenitor cell (OPC) recruitment and differentiation [3]. This hostile environment impedes remyelination and contributes to the progressive neurological decline seen in MS.

Oligodendrocyte dysfunction, characterized by the death of mature oligodendrocytes and impaired differentiation of OPCs, underlies the failure of effective remyelination in MS. Inflammatory mediators released by activated microglia and astrocytes, including TNF-α and reactive oxygen species, are toxic to oligodendrocytes and their precursors [4]. Single-cell RNA sequencing studies have revealed distinct subpopulations of oligodendrocytes and astrocytes in MS lesions, highlighting how maladaptive changes in glial cell states directly impair remyelination and drive lesion chronicity [5,6]. Thus, therapeutic strategies targeting the modulation of astrocyte reactivity and promotion of oligodendrocyte lineage progression are considered promising avenues for restoring CNS repair in MS.

S100B is a calcium-binding protein mainly concentrated in astrocytes in the nervous system. While low physiological concentrations (nM) of the protein are regarded to play a trophic role, at high toxic concentrations (μM), S100B is regarded as a Damage-Associated Molecular Pattern protein which triggers tissue reaction to damage in various disorders [7,8]. Its levels in biological fluids at present are currently employed as a reliable, even predictive, biomarker of active neural distress [9,10]. This concentration-dependent dichotomy underscores the dual role of S100B as both a physiologic trophic factor and a biomarker/potential mediator in the pathogenesis of various neurodegenerative disorders [11,12,13,14,15].

In this respect, high (micromolar) concentrations of S100B have been shown to participate in several processes associated with cellular damage. In particular, at micromolar concentrations, S100B increases reactive oxygen species (ROS) production in neurons, facilitates glutamate-induced neuronal death, induces the perturbation of lipid homeostasis and cell cycle arrest, up-regulates inducible nitric oxide synthase (iNOS), and promotes the release of nitric oxide (NO) and the NO-dependent death of neurons and glia. It also activates astrocytes through a pro-inflammatory, RAGE-dependent autocrine loop, up-regulates cyclooxygenase-2 (COX-2) expression in microglia, impairs oligodendrogenesis, and alters neuronal survival via NF-κB signaling [16,17,18,19,20,21,22,23,24]. These effects are largely mediated through the interaction of extracellular S100B with the Receptor for Advanced Glycation End-products (RAGE), which initiates downstream pro-inflammatory signaling cascades, including the activation of NF-κB and the subsequent induction of cytokines and inflammatory mediators. This S100B–RAGE axis has been implicated in various neuropathological conditions and represents a key mechanism in the amplification of neuroinflammation.

In addition, it has been shown that extracellular S100B protein exerts regulatory effects on inflammatory cells, neurons, astrocytes, microglia, and endothelial and epithelial cells, and that the transduction effects of RAGE (Receptor for Advanced Glycation End-products), the receptor of S100B, and also of S100A12, primarily impact inflammatory cells and neurons. In addition, other cell surface molecules capable of interacting with S100 members were identified, suggesting that RAGE might not be a universal receptor for S100B and/or that a single S100B protein might interact with more than one receptor [25,26]. Therefore, due to their high specialization, the S100B protein is multifunctionally involved in a variety of cellular processes, such as cell cycle regulation, cell growth, cell differentiation, and motility, depending also on the interaction with its receptor [27,28].

A number of correlative evidence proposes that S100B high levels may play a promoting role also in multiple sclerosis (MS) [29,30,31,32]. In this respect, we showed that in the relapsing–remitting experimental autoimmune encephalomyelitis (EAE) mouse MS model, the inhibitor of S100B activity, pentamidine (PTM), ameliorates clinical scores and neuropathologic-biomolecular parameters [29]; the finding was later confirmed also in the chronic EAE mouse model [32]. Likewise, in the chronic EAE model, arundic acid (AA, *ONO-2506*), an inhibitor of S100B synthesis, as well as a silencer of the S100B gene, induced lower severity compared to the vehicle-treated mice, by the evaluation of clinical scores and neuropathologic molecular analysis, particularly in the early phase of disease onset [30]. A significant reduction of astrocytosis, demyelination, immune infiltrates, and proinflammatory cytokine expression was observed when the expression of the S100B protein was impaired, indicating the participation of S100B in neuroinflammatory processes [30,32], reasonably as an astrocytic activity, since astrocytes are known to express and release S100B and are also regarded to be directly involved in MS processes [9,10,33]. The dual role of S100B mirrors the pathological environment of MS, where reactive astrocytes and other glial cells change from supportive to detrimental roles. Consequently, S100B and its signaling pathways represent promising targets for modulating astrocyte function and attenuating neuroinflammation in MS. As a consequence, slightly enlarging the field of investigation, in this work, we focused our attention on different populations of glial cells, which are currently regarded to express S100B or act through S100B stimulation [9,10].

## 2. Results

### 2.1. S100B Silencing Reduces Disease Severity

**Figure 1:**
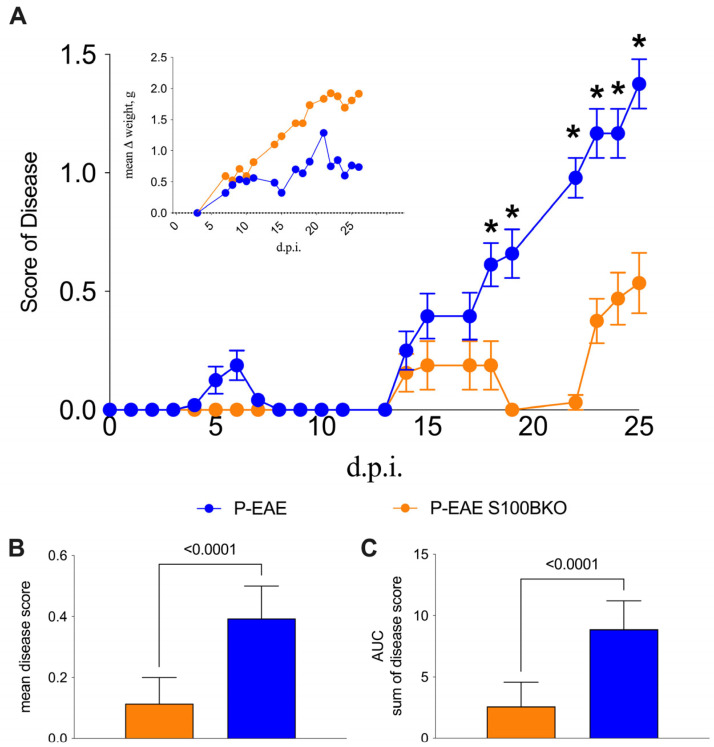
Clinical outcome of EAE in S100B KO versus WT mice. (**A**) represents disease scoring from 0 to 25 days post-immunization (d.p.i.). The inner graph describes the mean variation weight (g) in the monitoring days (d.p.i.). (**B**,**C**) display mean disease variation score and area under the curve (AUC) of disease score sum, respectively, in S100B KO (orange) versus WT mice (blue). Beneficial effects of *S100b* gene silencing were significant from day 18 to day 25. * = significant *p* values at different timepoints; In details: day 18 *, *p* = 0.006894; day 19 *, *p* = 0.000813; day 22 *, *p* < 0.000001; day 23 *, *p* = 0.000047; day 24 *, *p* = 0.000289; day 25 *, *p* = 0.000109.

The effect of the gene silencing of S100B on the clinical evolution of EAE in the C57Bl/6 MS model is displayed in Figure 1. The immunization of S100B KO and C57Bl/6 wild type (WT) mice produced significant differences in terms of clinical score (Mann−Whitney U test, *t*-test, and *p*-values are indicated in the figure legend); the KO group showed lower severity, when the mean and cumulative disease scores were compared to WT mice, particularly in the early phase of disease onset. Indeed, after day 18 post-immunization (d.p.i.), the group of WT mice (blue line and symbols) began to show symptoms that significantly persisted from day 18 to day 25. The KO mice (orange line and symbols) showed a delayed disease onset (certain mice never reached a disease score of 1) and led to a significantly milder disease. (Figure 1A). Moreover, the comparison of the average score (Figure 1B) and the cumulative disease (Figure 1C) of the two groups showed the significant differences between the KO and WT mice (both with a *p*-value < 0.0001, Mann−Whitney U test, *t*-test). Additionally, changes in body weight (Figure 1A) correlated with disease progression and were consistent with the observed clinical scores described by WT and S100B KO mice (Figure 1A).

### 2.2. S100B Silencing Impacts on Astrocytosis, Demyelination, and Microglia Activation

**Figure 2:**
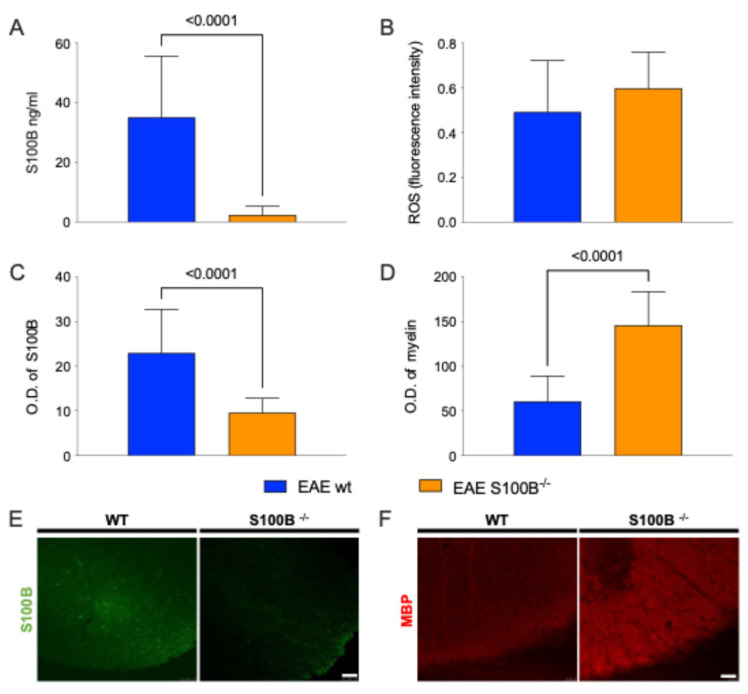
Evaluation of S100B in EAE-affected S100B KO (S100B^-/-^) and WT mice. (**A**) Bar graph reports ELISA assay of S100B protein (nanograms/milliliters). (**B**) Colorimetric assay of ROS is reported by bar graph. (**C**,**D**) Graphs show immunofluorescence semiquantification for S100B+ (**C**, **left**) and MBP+ areas (**D**, **right**) obtained from C1–C4 spinal cord slices. (**E**,**F**) Representative spinal cord (C1–C4) images show S100B (**E**, green signal) and MBP (**F**, red signal) from WT and S100B^-/-^ mice immunostainings. All the displayed results were analyzed by using Mann−Whitney *t*-tests among WT (blue) and S100B^-/-^ (orange). Scale bars: 75 µm.

ELISA analysis confirmed the absence of S100B protein in the brains of S100B KO mice compared to WT (*p*-value < 0.0001, Mann−Whitney U test; Figure 2A), verifying the inability of the KO strain to synthetize S100B.

We next examined oxidative stress levels in the CNS of the EAE-affected KO and WT mice. No significant differences were observed between the two groups (Figure 2B), indicating that S100B silencing does not markedly alter the overall oxidative balance.

To evaluate the impact of S100B gene deletion on gliosis and demyelination comparing EAE-affected KO and WT mice, we performed a semiquantitative analysis of correlated histological markers like S100B and Myelin Basic Protein (MBP) in the cervical spinal cord segments (C1–C4), regions known to exhibit pronounced pathological features in this EAE model (for a review, see [34]). Specifically, we quantified astrocytosis by measuring S100B-positive astrocyte staining, and demyelination by calculating the MBP-positive areas.

As expected, the silencing of S100B gene significantly reduced astrocytosis, as indicated by a lower number of S100B^+^ astrocytes in KO mice compared to WT (*p*-value < 0.0001, Mann−Whitney U test; Figure 2C,E). Moreover, demyelination was significantly attenuated in KO mice by preserving MBP^+^ areas (measured in Optical Density, O.D.; *p*-value < 0.0001, Figure 2D,F).

Representative 20× magnification images from the cervical spinal cord of one mouse per group are shown in Panels E–F of Figure 2, illustrating the observed differences in gliosis and myelin integrity.

**Figure 3:**
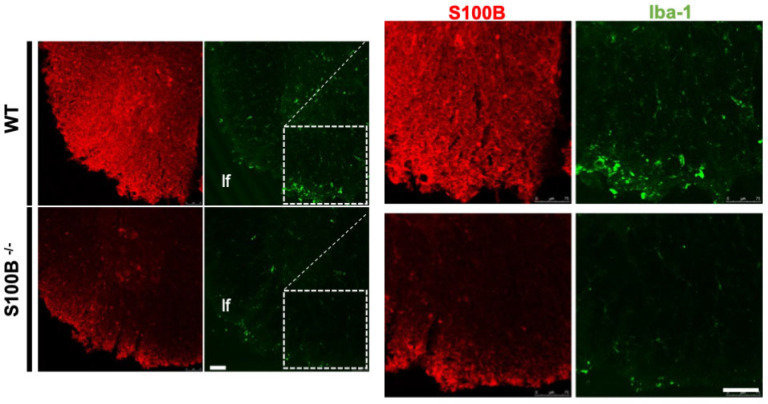
Evaluation of CNS S100B+ and Iba1+ cells in the cervical spinal cords (C1–C4) of EAE-affected WT and S100B KO mice. Representative confocal images were obtained from two different animals per group: WT (**top**) and S100B^-/-^ (**bottom**). Images show coronal sections of ventral cervical spinal cord. On the right, the insets highlight the lateral funiculus (lf) of the white matter providing a qualitative comparison of S100B (red) and Iba-1 (green) immunoreactivity. Scale bars: 75 µm.

To further support the clinical observations and explore whether S100B silencing can prevent, delay or reduce astrocytosis and microglial reactivity, we analyzed S100B and Iba1 expression in the same CNS regions of all mice. As expected, S100B signals were absent in KO mice. Notably, Iba1 immunoreactivity—indicative of microglial activation—was also reduced in the KO group, as shown by representative fluorescent images (Figure 3).

### 2.3. Pharmacological S100B Modulation Affects Definite Glial Cell Populations

Following the results obtained in the in vivo EAE model, we performed in vitro tests to confirm and deepen our understanding of the mechanisms involved in the counteraction of neuroinflammation. Based on previous studies demonstrating that in vivo modulation of S100B significantly affects astrocytic function [10,29,30] and considering our current results on the impact of S100B silencing in EAE-affected mice, we focused on definite cell populations of the CNS from EAE-affected WT mice. After the establishment of cell cultures of magnetically sorted neural subpopulations, cell viability and proliferation were assessed by using flow cytometric analysis.

**Figure 4:**
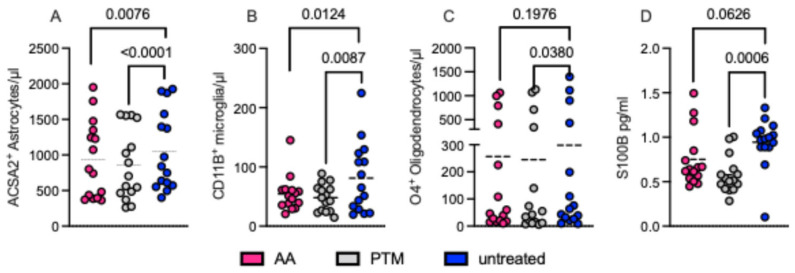
S100B modulation impacts on glial cell proliferation. Cell cultures of astrocytes, microglia, and oligodendrocytes were obtained by magnetically sorting cells from the CNS of EAE-affected WT mice (Disease score range: from 2 to 3, *n* = 15). After the generation of primary cell lines (10–14 days), cells were seeded (2 × 10^5^ in 100 μL) in quadruplicates. One-third of the wells were treated with AA (100 ng/mL, magenta), one-third were treated with PTM (100 ng/mL, gray), and the last third remained untreated (blue). After 24 h, cells were counted through flow-cytometry. (**A**) ACSA2^+^ for astrocytes; (**B**) CD11B^+^ for microglia; (**C**) O4^+^ for oligodendrocytes; CD31^+^ for endothelial cells; and CD45^+^/CD4^+^ for immune cells were used as negative controls for flow cytometry (see Supplementary Figure S1). After 24 h, cells were analyzed by flow cytometry, and supernatants were collected for S100B quantification by using ELISA (**D**). S100B levels are expressed in pg/mL. Statistical comparisons were performed using paired parametric *t*-tests.

We evaluated the effects of two pharmacological S100B inhibitors like AA and PTM on these cultures over a 24 h treatment period. Both inhibitors exerted their main effects on ACSA2^+^ astrocytes (*p* = 0.0076 and *p* < 0.0001, respectively) and *CD11B^+^* microglia (*p* = 0.0124 and *p* = 0.0087, respectively). A milder but still notable effect was observed on O4^+^ oligodendrocytes (AA: *p* = 0.1976; PTM: *p* = 0.038). Thus, the comparison of both S100B inhibitors revealed similar impacts on all cell cultures, although with different efficiencies (Figure 4).

To evaluate the milieu of cells in vitro during S100B inhibition, we focused in particular on AA, which reduces S100B synthesis. Thus, we analyzed protein and mRNA expression levels of S100B and TNFα, an inflammatory cytokine usually produced by astrocytes during inflammatory stimulation, in the supernatants of astrocytic cultures through ELISA and q-RealTime-PCR (q-RT-PCR).

**Figure 5:**
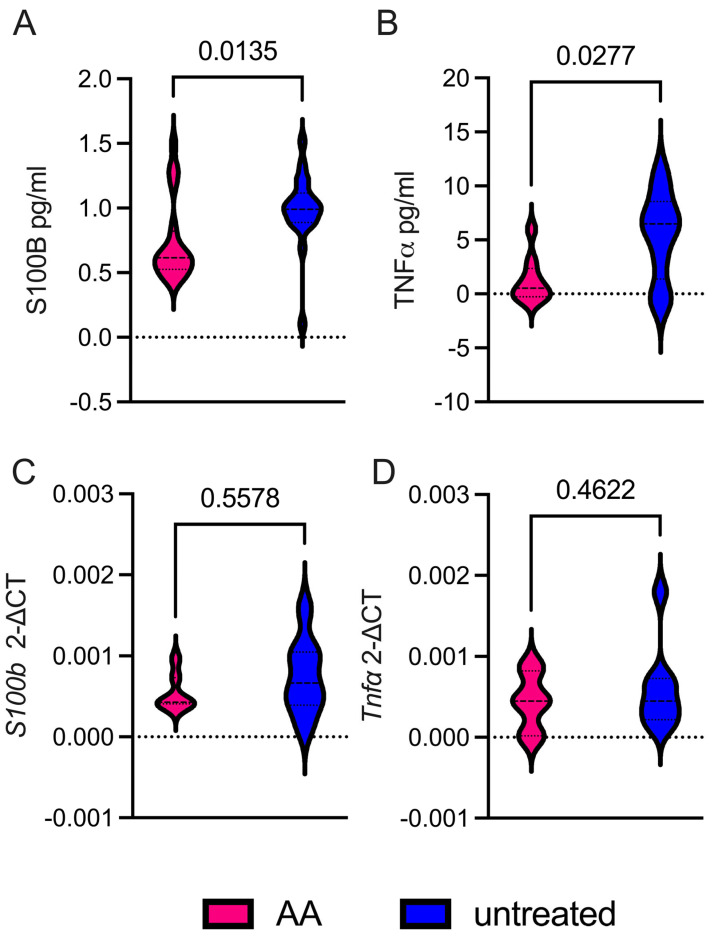
Arundic acid (100 ng/mL) modulates astrocytic S100B and TNFα release via post-transcriptional mechanisms in vitro. (**A**,**B**) Violin plot graphs report ELISA assay results of S100B and TNFα protein levels, respectively (expressed as picograms/milliliters) in the supernatants of astrocytic cultures; AA treated (fuchsia) and untreated (blue). (**C**,**D**) Graphs display q-RT-PCR results of *Tnfα* (**C**) and *S100b* (**D**) gene expression levels (expressed as 2^−∆CT^). Data results have been analyzed by using paired *t*-tests among mouse groups. The mean values and standard deviations were calculated from 20 independent samples, each obtained from a different animal (*n* = 20). To directly assess the role of EAE astrocytic S100B, we measured the cell vitality in glial cell types isolated from EAE S100B KO and WT mice. The ablation of S100B reduced the number of glial cells, selectively impacting astrocyte and oligodendrocyte subpopulations in different ways. Firstly, we compared, through flow cytometry, astrocyte subpopulations defined as CD45^-^/CD31^-^/CD11B^-^/O4^-^/ACSA2^+^/GLAST^+^ (namely ACSA2+, Figure 6A) and CD45^-^/CD31^-^/CD11B^-^/O4^-^/ACSA2^-^/GLAST^+^ (namely ACSA2^-^, Figure 6B). These subpopulations were compared between WT and S100B KO mice affected by EAE. ACSA2^+^ cells were consistently more abundant and viable in EAE-affected S100B KO than WT mice (*p* = 0.0741, Figure 6A), while the ACSA2^-^ subpopulation was significantly enriched compared to controls (*p* = 0.019, Figure 6B). This marker is recognized as a marker of progenitors, with astroglia-like characteristics able to generate mainly astrocytes and oligodendrocytes [35]. Reduced ACSA2 expression in GLAST^+^ cells may indicate a lineage commitment shift, either during development or in response to disease conditions.

Following 24 h of AA treatment, we observed a significant reduction in the concentrations of S100B and TNF-α in the supernatants (Figure 5A,B, *p* = 0.0135 and *p* = 0.0277, respectively). In contrast, quantitative analysis of mRNA levels revealed no statistically significant differences in the expression of S100B or TNF-α following AA treatment (Figure 5C,D).

These results suggest that AA in vitro treatment may exert post-transcriptional regulatory effects on the release of astrocyte-derived S100B and TNF-α under inflammatory conditions (Figure 5C,D, ns).

**Figure 6:**
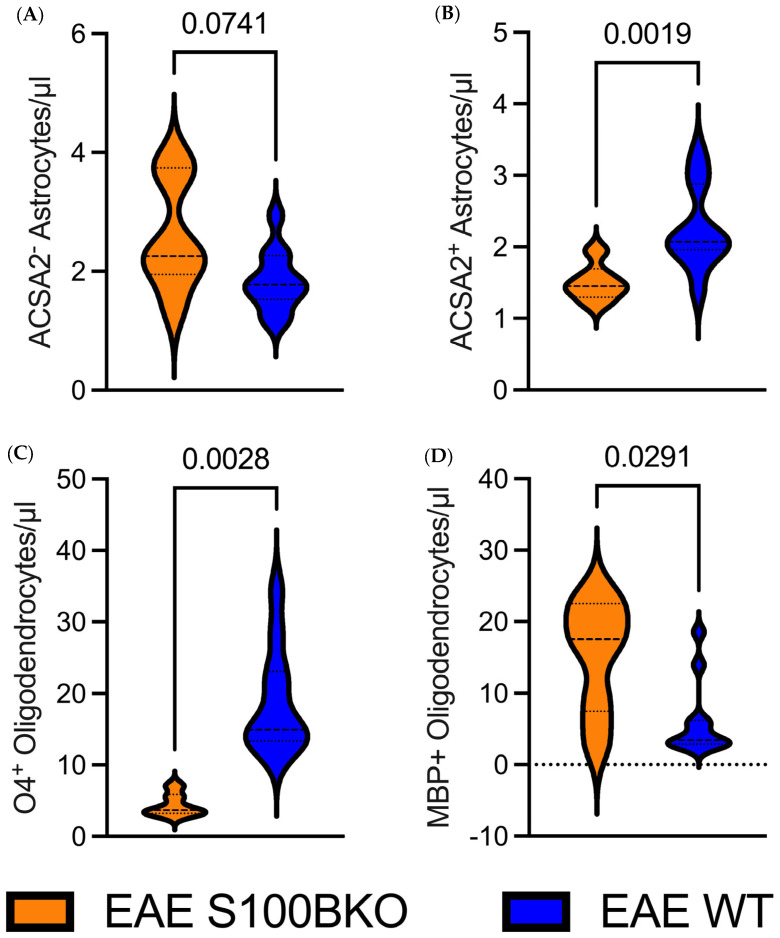
Astrocytic S100B ablation alters glial cell composition and promotes oligodendrocyte maturation and myelin preservation in the EAE condition. (**A**,**B**) ACSA2^+^ astrocyte survival is significantly reduced in the absence of S100B (A, *p* = 0.0741), while ACSA2^-^ astrocyte vitality is consistently reduced (B, *p* = 0.0019). (**C**,**D**) Oligodendrocytes during different phases of proliferation (expressing O4^+^ and MBP^+^, respectively) are significantly reduced in the absence of the S100B gene.

We next examined oligodendrocyte subpopulations. The analysis of CD45^-^/CD31^-^/CD11B^-^/ACSA2^-^/GLAST^-^/O4+ cells (O4+ cells), which are regarded as oligodendrocyte precursor cells (OPCs), showed these cells to be significantly less abundant in S100B KO mice than in WT (*p* = 0.0028, Figure 6C). In contrast, mature oligodendrocytes, identified as CD45^-^/CD31^-^/CD11B^-^/ACSA2^-^/GLAST^-^/MBP+ (MBP^+^ cells), were significantly more abundant in the S100B KO group than in WT mice (*p* = 0.0291, Figure 6D). These findings suggest a possible role of S100B in the balance of demyelination/remyelination. These results were in line with the MBP immunofluorescence analysis of cervical spinal cord segments (C1–C4) in tissue sections from EAE-affected WT and S100B KO mice (Figure 3), where the quantitative analysis of the fluorescent signal confirmed a significant difference in terms of demyelinated areas between the two groups (*p* < 0.001, *n* = 6, Figure 3 and Supplementary Figure S1).

## 3. Discussion

The present study provides evidence supporting the pivotal key role played by the S100B protein, as a constituent of definite glial cell types, in MS processes and their experimental models already proposed by other studies [29,30,31,32]. As expected, poor S100B reactivity could be observed in astrocytes or oligodendrocytes derived from S100B-ablated mice (for reviews, see [9,10]). In particular, in vivo analyses obtained in S100B KO mice supported the hypothesis that the silencing of the S100B gene is efficient during the onset of MS, behaving as a disease-delaying factor (Figure 1 and Figure 2). It should be noted, in this respect, that the fluorescent method used might give an account of the results obtained on ROS, directly examined here for the first time in S100B KO mice, and otherwise shown to be regulated by S100B in various pathological conditions and known to be active in MS processes (for a review, see [36,37]). One might consider, in this respect, that despite the establishment of oxidative stress as a crucial process in MS development and progression, ROS scavengers have had limited success in animal studies, as in the present study (for a review, see [22]). In addition, since the silencing of S100B may not achieve complete ablation of the protein, a partial knockdown could result in very low residual S100B levels, sufficient to maintain its functional roles, including modulation of oxidative stress responses. It is therefore not possible to predict whether the lack of reduced ROS production in S100B KO mice is due to interactions with other regulatory molecules or post-translational phenomena that could be somehow able to mask the effect of gene silencing on oxidative stress pathways [38,39]. Furthermore, discrepancies with previous findings may reflect differences in cellular context, stress conditions, or compensatory mechanisms following gene silencing. Additionally, S100B is known to interact with various signaling pathways beyond those directly related to oxidative stress [40]. For instance, S100B has been implicated in the regulation of calcium homeostasis, gene expression, and neuroinflammatory responses, as highlighted also by our results. For instance, it has been reported that S100B KO mice could have a reduced serotonergic activity limiting serotonergic neurons in culture [41,42]. These pathways could compensate for the loss of S100B in the context of oxidative stress, thereby masking potential effects on ROS levels. To address these possibilities, future studies could employ more specific and sensitive assays for ROS detection, such as those targeting mitochondrial ROS or lipid peroxidation products that was not the focus of our report. Additionally, confirming the extent of S100B knockdown through quantitative protein analysis would provide clarity on the functional consequences of its silencing.

Furthermore, gene ablation of S100B significantly decreased microglial activation, as indicated by a qualitative reduced IBA1 expression (Figure 3), which is known to be upregulated during microglia activation [43,44]. These results suggest a putative role of S100B in modulating microglial activity during EAE processes (Figure 3), indicating a possible involvement of this protein in glial reactivity that warrants further dedicated investigation.

In addition, pharmacological inhibition or genetic ablation of S100B also impaired the proliferation and survival of S100B-expressing glial cells, primarily astrocytes and, even if to a lesser extent, oligodendrocytes (for reviews, see [9,10]). These findings indicate that this protein could be involved in important functions within these cells. Notably, microglial proliferation was similarly affected (Figure 4). The reduction observed with pentamidine treatment, a known S100B antagonist, aligns with the recognized stimulatory role of S100B on microglial function (for a review, see [45]). However, the direct effect of AA, which inhibits S100B synthesis, deserves interpretation, since the expression of S100B in microglial cells is debated ([45,46]).

Our data suggest that AA may act on S100B at a post-transcriptional level (Figure 5). In this respect, one possible explanation could be that since cells are isolated from EAE mice, which are known to overexpress S100B [29,30,32], microglia retain extracellular S100B secreted by astrocytes, which is then decreased by AA treatment [47]. Supporting this interpretation, previous findings have shown that AA can inhibit the release of the inflammatory cytokine TNF-α in isolated astrocytes (Figure 5), which is consistent with the proposed pro-inflammatory role of S100B in these cells [9,10,29,30]. Similarly to what was previously hypothesized for oxidative stress in S100B KO mice, the treatment with AA is able to block mRNA of S100B, and this mechanism could also explain that mRNA expression levels of TNF-α were discrepant when compared to the protein levels. Moreover, distinct samples were evaluated with two distinct techniques, ELISA and PCR assays. TNF-α and S100B protein levels were evaluated in the supernatants of cells after 24 h of inhibition with AA finding significantly different expression. After 24 h, we tested also mRNA derived from cells, finding no changes comparing unstimulated and inhibited samples, possibly because the inhibition of AA at this timepoint on the brain-derived cells were not able to permanently block mRNA. Therefore, in the absence of such stimuli, silencing S100B may not significantly impact the redox state and inflammation, suggesting that its contribution to oxidative stress and neuroinflammation is conditional rather than constitutive.

In pathological contexts, such as MS, reactive gliosis leads to elevated S100B levels, which can differentially impact glial subpopulations. It has been widely demonstrated that high extracellular concentrations of S100B impair glial cell survival, partly through activation of the Receptor for Advanced Glycation End-products (RAGE) and subsequent induction of oxidative stress and inflammation [48,49,50,51]. In our model, it may be noteworthy that gene ablation of S100B did not affect microglia survival—as assessed via CD11b (for a review, see [52]) and Iba1 (for a review, see [45]) cell surface biomarkers—but significantly reduced survival of astrocytes and oligodendrocytes (Figure 6), which is undoubtably expressed in WT mice (for reviews, see [9,10]).

It may be speculated that S100B may contribute, directly or indirectly through astrocytosis, to the vulnerability of specific oligodendrocyte subtypes in a demyelinating context such as EAE. A deeper understanding of the context-specific roles of S100B in oligodendrocyte biology may uncover novel therapeutic strategies aimed at promoting remyelination through modulation of astrocyte–oligodendrocyte interactions. If this hypothesis is validated in MS patients, counteracting S100B could be particularly useful during the remission phases of the disease, potentially contributing to remyelination processes.

Taken together, this evidence offers useful information to better clarify the role played by S100B protein in MS-related neuroinflammation, delineate the specific glial subtypes involved, and support the rationale for targeting S100B in therapeutic strategies. The potential for clinical translation of our data to humans lies precisely in the possible therapeutic window. One of the unmet needs in the treatment of MS and in particular RR-MS is the prolongation and maintenance of the remission state for as long as possible, with the possibility of being able to recover at this stage of the disease from most disabilities. The inhibition of S100B, if also confirmed in humans, could have its use precisely in this therapeutic window by favoring recovery with remyelination and counteracting neuroinflammation. This scenario proposes S100B as a therapeutic target for MS, as well as for different neural disorders which appear to share some pathogenic features, reasonably attributable to neuroinflammation supported by astrocyte activation [9,10].

### Limitations of the Study

Despite the valuable insights provided by this study, several limitations should be acknowledged. First, while gene ablation and pharmacological inhibition of S100B yielded significant effects on glial cell behavior and MS-related processes, the extent of S100B knockdown was not quantitatively verified, leaving uncertainty about residual protein levels and their potential functional impact. This is particularly relevant given the possibility that even low levels of S100B might retain biological activity, especially in modulating oxidative stress and neuroinflammation. Additionally, the fluorescent ROS detection method employed may lack the specificity and sensitivity needed to fully capture the complexity of redox changes in S100B-deficient models, possibly masking subtle but biologically relevant effects.

Another important limitation lies in the use of the EAE model, which, while informative, may not fully replicate the heterogeneous pathology of human MS, particularly in the context of the relapsing–remitting dynamics and remyelination phases. Furthermore, while S100B ablation affected astrocyte and oligodendrocyte viability, its effects on microglia were less conclusive, raising questions about the differential roles of S100B across glial subtypes and potential compensatory mechanisms. Finally, the study did not address possible off-target effects of the pharmacological agents used, particularly arundic acid (AA), which may exert broader effects beyond S100B suppression. These limitations suggest that further studies using refined models, quantitative knockdown validation, and more targeted assays are needed to clarify the precise role of S100B in MS pathogenesis and to evaluate its therapeutic potential.

## 4. Materials and Methods

### 4.1. Animal Procedure

The induction of EAE was performed on two different strains of female mice (8–10-week-old): C57Bl/6 mice (namely wild type, WT mice) purchased from Charles River (USA) and B6Brd, B6N-Tyr<c-Brd> S100b<tm1a(EUCOMM)Wtsi>/WtsiCnbc (namely S100B KO) purchased from the EMMA node at CNB-CSIC, Centro Nacional de Biotecnologia (RRID:IMSR_EM:05301, Madrid, Spain). The conditions for induction and management of EAE followed the procedures described in our previous works [30,53,54]. Briefly all mice were injected with a peptide derived from myelin oligodendrocyte glycoprotein (MOG_35–55_, 50 mg/mL; Sigma-Aldrich, S.r.l., Milan, Italy) emulsified with a *Mycobacterium tuberculosis* (strain H37Ra, ATTC 25177)-derived adjuvant (complete Freund’s adjuvant (CFA), Sigma-Aldrich S.r.l., Milan, Italy), 4 mg/mL. To enhance the neuroinflammation allowing the breakage of the blood–brain barrier, two doses of *Bordetella pertussis* toxin (BDT) (Sigma-Aldrich S.r.l., Milan, Italy) were intraperitoneally administered (150 ng/mice × 2 times, at day 0 and 48 h after EAE induction). As a control of the procedure, different mice from both groups were injected with an emulsion of CFA and PBS (vehicle). The mice were monitored daily for body weight variations and for clinical signs and symptoms (CSS) development, as described in [55,56,57,58].

WT and S100B mice were randomly distributed into four different groups, vehicle-WT healthy controls (CTRL-WT, *n* = 12), vehicle-S100B-KO healthy controls (CTRL-KO, *n* = 12), EAE-induced WT (EAE-WT, *n* = 24), and EAE-induced S100B KO (EAE-KO + AA, *n* = 24), in four distinct experiments. Ethical guidelines were followed as foreseen by Italian laws for animal welfare and all procedures were approved by the Italian Ministry of Health (authorization *n* = 15/2021-PR protocol 1F295.120).

The mice were sacrificed through perfusion with PBS, after deep intraperitoneal anesthesia (87.5 mg/kg ketamine and 12.5 mg/kg xylazine, 0.1 mL/20 g body weight). Brain tissues were harvested, subdivided into two half-brains (right and left), and then dissected into distinct areas: cerebellum, cortex (rostral area), and spinal cords. Samples from each half-brain were immediately thawed and stored at −80 °C for molecular analysis by q-PCR, ELISA, and quantization of ROS and NOS; the other half-brains were preserved for subsequent in vitro assays. Spinal cords were also harvested and immersed in a fixative solution of 4% paraformaldehyde (PFA) and then incubated for immunofluorescence analyses [30].

### 4.2. Astrocytes’ Primary Isolation

After sacrifice, the tissue was dissociated into single-cell suspensions following the manufacturer’s instructions of the mouse and rat Adult Brain Dissociation Kit (Milteny Biotec, Bergisch Gladbach, Germany, 130-107-677). Later, the cell suspensions were prepared for the magnetic separation by the Astrocytes Cell Surface Antibody 2 Microbead (ACSA2) (Milteny Biotec, Germany, 130-097-678). Following the manufacturer’s instructions, we set the experiment for 2 × 10^7^ cells number. After this we obtained two cell populations: astrocytes ACSA2+ and the negative fraction composed of oligodendrocytes, microglial cells, and astrocytes ACSA2-. Magnetic sorting was performed on a total of 20 samples derived from the brains of EAE-affected mice (WT, *n* = 10, and KO, *n* = 10) from three distinct experimental sets obtained from a minimum of 1 × 10^6^ to a maximum of 1.5 × 10^6^ total cells for each glial subpopulation.

### 4.3. Cell Cultures

An aliquot of ACSA2^+^ and ACSA2^-^ subpopulations (2 × 10^5^ cells) from each EAE-affected mouse (WT *n* = 10 and KO *n* = 10) was previously analyzed by flow cytometry to verify purity and cell viability of different glial cell subpopulations.

Both glial cell subpopulations from each EAE-affected mouse (WT, *n* = 10; KO, *n* = 10) were then seeded onto 6-well tissue culture plates previously pretreated with Poly-L-lysine (EMD Millipore Corp., Burlington, MA, USA, A-005-C) and Laminin (Sigma, UK, L2020) at a concentration of 1 × 10^6^ cells/well. Cells were maintained in culture for 25 days using Dulbecco’s Modified Eagle’s Medium/Ham’s F-12 without L-Glutamine (DMEM F12) (Euroclone, Pero, Italy, ECM0090L) implemented with Fetal Bovine Serum (FBS) (Euroclone, Italy, ECS0180L), L-Glutamine (Euroclone, Italy, ECB3000D), Penicillin–Streptomycin (Euroclone, Italy, ECB3001D), and Amphotericin B (Euroclone, Italy, ECM0009D). Next, glial cells were detached by enzymatic action of Trypsin-EDTA (Euroclone, Italy, ECB3052D). An aliquot of each cell population (1 × 10^5^ cells) derived from each EAE mouse was evaluated through flow cytometric analyses for cell viability of the different cell subsets as described below. The remaining cells were seeded in 3 distinct Poly-L-lysine/Laminin pretreated 96-well TC plates (in sextuplicate for each sample, 1 × 10^5^ cells/well). For each sample, 2 wells were treated with a vehicle (PBS), 2 wells were treated with AA (100 ng/mL), and 2 wells were treated with PTM (100 ng/mL). In this way, ACSA2^+^ and ACSA2^-^ subsets derived from each KO/WT mouse were exposed for 24 h in duplicates for each condition (vehicle/AA/PTM). After 24 h cultures/treatments, the supernatants were recovered and preserved for molecular analyses, while cells were in part analyzed through flow cytometry and in part preserved for molecular analyses.

### 4.4. Flow Cytometry Assay

To evaluate the viability of freshly extracted brain cells and the effect of 24 h treatments of AA and PTM on astrocytes ACSA2+ and the population of ACSA2-, we performed a flow cytometry assay using Cytoflex (Beckman Coulter, Brea, CA, USA) before and after treatments. The cells were marked in different combinations with the following anti-mouse antibodies: CD45-PerCP (BD biosciences, Franklin Lakes, NJ, USA, 561047), GFAP-PE (Milteny Biotech, Bergisch Gladbach, Germany, 130-118-351), ACSA-2 PE-Vio^®^615 (Milteny Biotech, Germany, 130-116-146), CD31-Vio^®^Bright (Milteny Biotech, Germany, 130-111-358), CD11b-VioGreen^TM^ (Milteny Biotech, Germany, 130-113-811), O4-APC (Milteny Biotech, Germany, 130-119-897), GLAST (ACSA-1) PE (Milteny Biotech, Germany, 130-118-344), MBP-APC (Bio-techne, Minneapolis, MN, USA, NBP2-22121APC), and DAPI Staining Solution (Milteny Biotech, Germany, 130-111-570) following the respective manufacturer’s instructions. Analysis and plotting were performed using the Kaluza software 3.0 (Beckman Coulter, Brea, CA, USA) as described in Supplementary Figure S2. Briefly, the gating strategy consisted of a preliminary FSC/SSC plot to exclude debris. Then, the plotting of CD45/CD3 allowed us to exclude infiltrating immune cells gating CD45-/CD3- glial cells (Supplementary Figure S2B). We successively built on this gate three distinct plots for the detection of ACSA2+, CD11B+, and O4+ cells (Supplementary Figure S2C, Figure S2D and Figure S2E, respectively). On these plots we gated Astrocytes, Microglia, and Oligodendrocytes, all evaluated with DAPI to reveal cell viability on gated subpopulations (Supplementary Figure S2F, Figure S2G, and Figure S2H, respectively). The analysis was performed evaluating the percentages and concentrations (number/mircoL) of the distinct glial subpopulations.

### 4.5. RT-qPCR Assay

Brain areas, collected as described above, were lysed through mechanical and enzymatic homogenization. Total RNA was extracted with an RNeasy mini kit following the manufacturer’s instructions (Qiagen, Hilden, Germany). The cDNA obtained (sensiFast cDNA synthesis kit, Meridian Bioscience, Cincinnati, OH, USA) was used to quantify the expression level of *Tnfα* and *S100b* in RT-qPCR, using an IQ SYBR^®^ Green supermix and iQ5 Multicolour Real Time PCR Detection System (Biorad, Hercules, CA, USA). *β-actin* was used as housekeeping. The 5′-3′ primer sequences were as follows: *β-actin* forward CGTAAAGACCTCTATGCCAACA; *β-actin* reverse GGAGGAGCAATGATCTTGATCT; *Tnfα* forward CGAGTGACAAGCCTGTAGCCC; *Tnfα* reverse GTCTTTGAGATCCATGCCGTTG [59]; *S100b* forward AAAGGCTCATGGGCTCGAAG; and *S100b* reverse GAAGGGGGTTGGGGTTTCAT. The procedures were described in our previous works [29]. Relative mRNA expression levels were calculated by normalizing on *β-actin*, using the 2^−ΔCt^ method. Similarly to flow cytometry, the sample sizes evaluated for mRNA expression levels was on the total samples derived from in vivo (*n* = 36, WT = 12 and S100BKO = 24) and in vitro experiments (*n* = 20; WT = 10 and S100BKO = 10). All the analyses of each gene/primer were repeated in triplicate, and then the mean of each sample was normalized with the mean of the housekeeping gene levels, also performed in triplicate.

### 4.6. S100B ELISA Assay

S100B and TNFα were quantified in the half-brain-derived homogenates of EAE-affected mice and from supernatants of cultured cells by using a SimpleStep ELISA^®^ kit (Abcam, Cambridge, UK, ab285283 and ab208348). Both kits were used according to the manufacturer’s instructions. S100B and TNFα were evaluated by semi-quantitative analysis on brain-derived homogenates of EAE mice and on the above-described cell culture-derived supernatants, comparing cell cultures treated with vehicle with the ones treated with AA. The sample size for this technique was based on the total samples derived from in vivo (*n* = 36, WT = 12 and S100BKO = 24) and in vitro experiments (*n* = 20; WT = 10 and S100BKO = 10).

### 4.7. Assessment of Intracellular Reactive Oxygen Species (ROS)

ROS levels were measured in tissue homogenates using the OxiSelect™ Intracellular ROS Assay Kit (Green Fluorescence; Cell Biolabs, Inc., San Diego, CA, USA), according to the manufacturer’s instructions. The assay employs the cell-permeable probe DCFH-DA, which is deacetylated to a non-fluorescent intermediate and then oxidized by ROS to generate the fluorescent compound DCF.

Tissue samples were homogenized and incubated with DCFH-DA at 37 °C for 30–60 min. After incubation, fluorescence was measured at 480 nm excitation and 530 nm emission using a microplate reader. ROS levels were quantified based on fluorescence intensity and normalized to homogenate total protein content, determined with the Bio-Rad Protein Assay (Bio-Rad Laboratories, Hercules, CA, USA).

### 4.8. Immunofluorescence and Data Analysis

Following mouse sacrifice, spinal cords were dissected, post-fixed in 4% paraformaldehyde for at least of 48h, and transferred to a 30% sucrose solution. Subsequently, they were frozen at −80 °C for a long storage period before processing. Only cervical spinal cord tissues were additionally dissected and embedded in O.C.T. KIjilli compound and coronal-sectioned in 30 µm thickness slices with Leica Cryostat. Then, sections were incubated with primary antibodies diluted in 0.3% Triton X-100 permeabilization solution for 48 h on 4 °C bascule. The following antibodies were used: fluoromyelin red (1:200, ThermoFisher (Waltham, MA, USA)), S100B (1:600, Synaptic System (Göttingen, Germany)), and Iba1 (1:500, NovusBio (Centennial, CO, USA)). Secondary reaction was made with coupled anti-chicken Alexa Fluor 488 and anti-goat antibodies (1:200, ThermoFisher). Fluorescent images were obtained by confocal microscopy (Leica (Wetzlar, Germany)), with 20x magnification. Myelination and S100B protein levels were detected through optical density (O.D.) analysis by using a predetermined area (ROIs, 180 × 180 µm approximately). From each acquired image, four random ROIs were used for fluorescence analysis.

### 4.9. Quantification and Statistical Analyses

All quantitative data were expressed as the mean ± standard deviation (SD) or standard error of the mean (SEM), as indicated in the figure legends. In detail, the Figure 1 graph shows values of clinical scores and symptoms (CCS) in EAE mice evaluated as described in the Materials and Methods. The data report the average clinical score for EAE, comparing WT and S100B-KO mice using unpaired *t*-tests assuming that both groups of samples were from populations with the same SD and applying the two-stage linear step-up procedure of Benjamini, Krieger, and Yekutieli, as recommended by the analysis software, in order to compare the difference of each day after immunization. For Figure 1B,C and Figure 2 the comparisons were performed using unpaired nonparametric *t*-tests and Mann–Whitney tests. All values are given as means ± SDs. The statistical analysis for Figure 4, Figure 5 and Figure 6 consisted of paired *t*-tests and one-way analyses of variance (ANOVAs), followed by Tukey’s multiple comparison post hoc tests. All the statistical analyses were performed using Graphpad Prism v10.4.2.

## Data Availability

Data is contained within the article.

## Supplementary Materials

The following supporting information can be downloaded at: https://www.mdpi.com/article/10.3390/ijms26135948/s1.
