# Supplementary material for: Effect on Different Glial Cell Types of S100B Modulation in Multiple Sclerosis Experimental Models
Source: Int J Mol Sci. 2025 Jun 20;26(13):5948. doi: 10.3390/ijms26135948 (PMC12249851; doi:10.3390/ijms26135948)
Supplement: Supplementary file 1 [file ijms-26-05948-s001.zip › KO S100B supplementary figures.pdf]

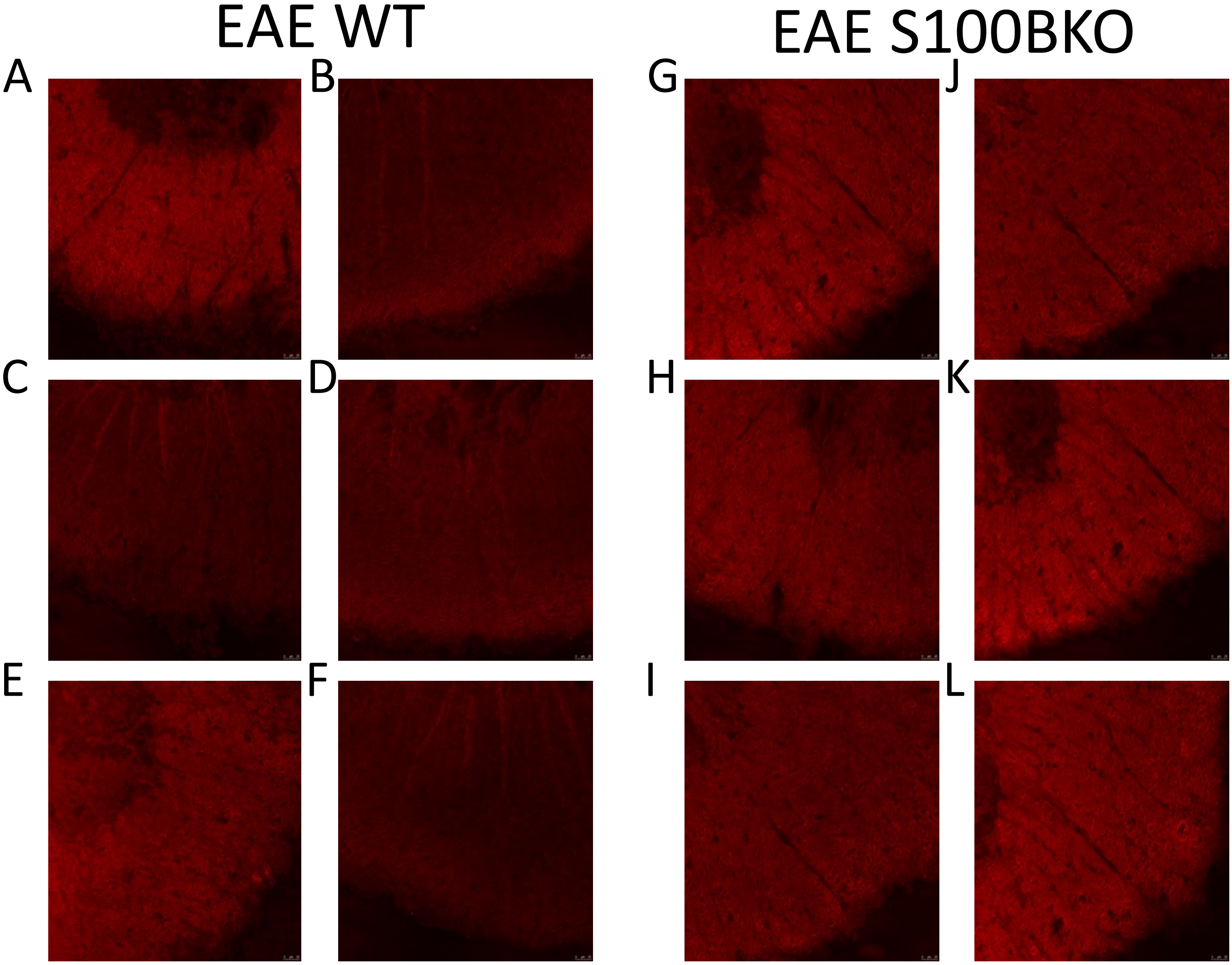

Supplementary Figure S1. S100B ablation preserves oligodendrocyte maturation and myelin production during EAE. A-L immunofluorescence and data analysis that display a significantly reduced myelin in the group of EAE affected WT mice (C1-C4 regions) when compared to EAE affected S100B KO mice (C1-C4 regions). The quantification is displayed in figure 3.

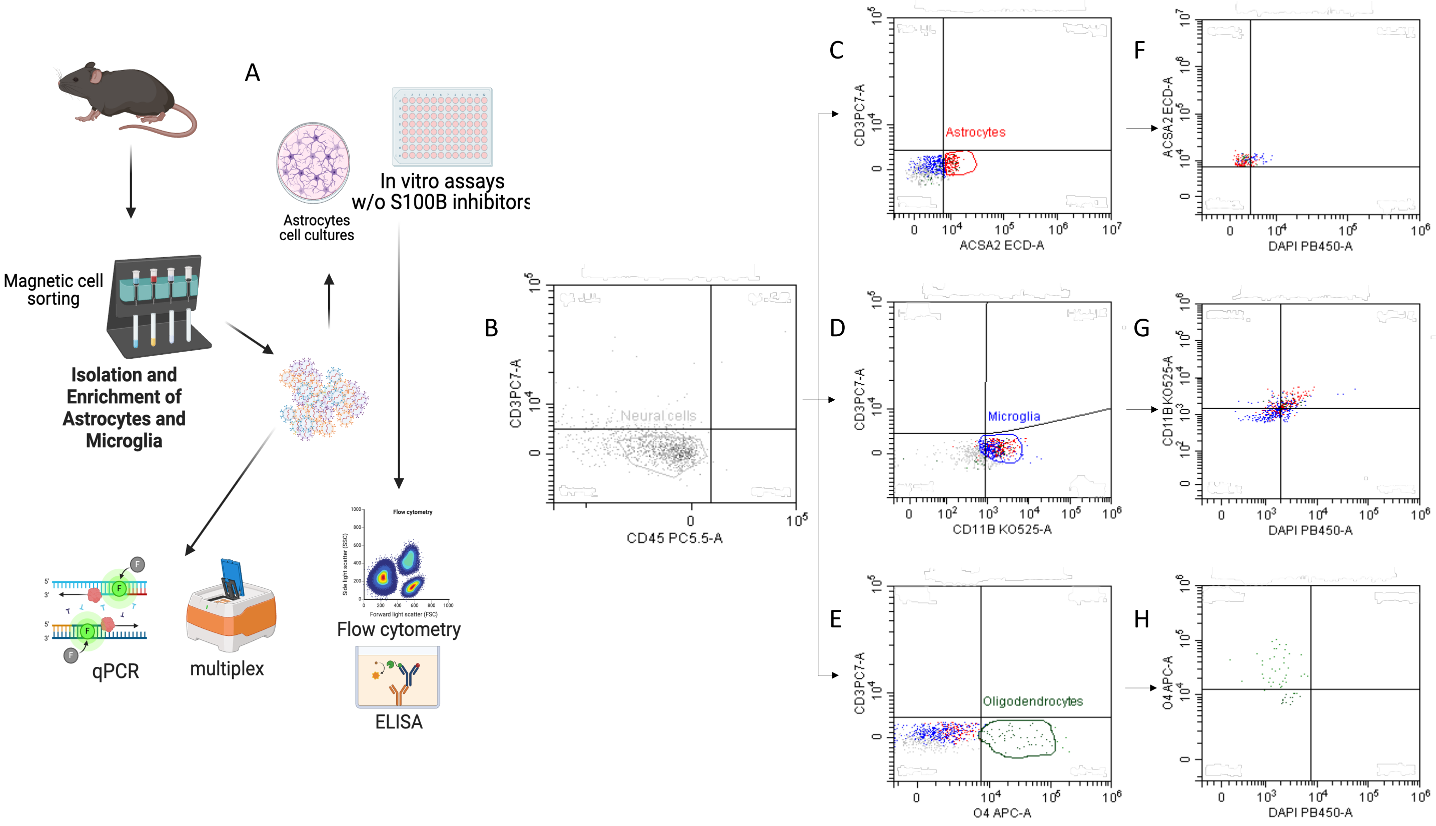

Supplementary Figure S2. Research plan and flow cytometry workflow. A. Cell cultures of Astrocytes, Microglia and Oligodendrocytes were derived from CNS of EAE affected WT and KO-S100B mice (score between 2 and 3, n=20) and magnetically sorted. After the generation of primary cell lines (10-14 days), cells were seeded ($2x10^5$ in 100 µl) in quadruplicates. Half wells were treated with AA and half remained untreated. After 24 h cells were counted through flow-cytometry. Collected supernatants were next analyzed by ELISA for S100BComparisons were performed through paired parametric t test. **B.** The Dot Plot excludes eventual infiltrating cells (CD45+ and/or CD3+). The gate includes cultured neural glial cells. **C, D and E.** The gate in C includes astrocytes (ACSA-2+ cells, in red); in D the gate shows microglial cells (CD11B+, in blue); in E the gate displays Oligodendrocytes cells (O4 APC, in green). **F, G and H.** Dot plots displays dead cells DAPI+) in all previously described subpopulations.
